# Mapping the topographic epitope landscape on the urokinase plasminogen activator receptor (uPAR) by surface plasmon resonance and X-ray crystallography

**DOI:** 10.1016/j.dib.2015.08.027

**Published:** 2015-09-04

**Authors:** Baoyu Zhao, Sonu Gandhi, Cai Yuan, Zhipu Luo, Rui Li, Henrik Gårdsvoll, Valentina de Lorenzi, Nicolai Sidenius, Mingdong Huang, Michael Ploug

**Affiliations:** a State Key Laboratory of Structural Chemistry, Fujian Institute of Research on the Structure of Matter, Chinese Academy of Sciences, Fuzhou, Fujian 350002, China; b FIRC Institute of Molecular Oncology, Via Adamello 16, 20139 Milan, Italy; c Finsen Laboratory, Rigshospitalet, Copenhagen Biocenter, Ole Maaløes Vej 5, DK-2200 Copenhagen N, Denmark; d Biotech Research and Innovation Centre (BRIC), Copenhagen Biocenter, Ole Maaløes Vej 5, DK-2200 Copenhagen N, Denmark; e Danish-Chinese Centre for Proteases and Cancer

**Keywords:** uPAR, CD87, Epitope mapped antibodies, SPR, Allostery, Hot spots, Vitronectin, Cancer invasion

## Abstract

The urokinase-type plasminogen activator receptor (uPAR or CD87) is a glycolipid-anchored membrane protein often expressed in the microenvironment of invasive solid cancers and high levels are generally associated with poor patient prognosis (Kriegbaum et al., 2011 [1]). uPAR is organized as a dynamic modular protein structure composed of three homologous Ly6/uPAR domains (LU).This internally flexible protein structure of uPAR enables an allosteric regulation of the interactions with its two principal ligands: the serine protease urokinase-type plasminogen activator (uPA) and the provisional matrix protein vitronectin (Vn) (Mertens et al., 2012; Gårdsvoll et al., 2011; Madsen et al., 2007 [2–4]). The data presented here relates to the non-covalent trapping of one of these biologically relevant uPAR-conformations by a novel class of monoclonal antibodies (Zhao et al., 2015 [5]) and to the general mapping of the topographic epitope landscape on uPAR. The methods required to achieve these data include: (1) recombinant expression and purification of a uPAR-hybrid protein trapped in the desired conformation [patent; WO 2013/020898 A12013]; (2) developing monoclonal antibodies with unique specificities using this protein as antigen; (3) mapping the functional epitope on uPAR for these mAbs by surface plasmon resonance with a complete library of purified single-site uPAR mutants (Zhao et al., 2015; Gårdsvoll et al., 2006 [5,6]); and finally (4) solving the three-dimensional structures for one of these mAbs by X-ray crystallography alone and in complex with uPAR [deposited in the PDB database as 4QTH and 4QTI, respectively].

**Specifications table**

**Table:** 

Subject area	Protein structure and biochemistry
More specific subject area	Trapping a flexible protein structure in a defined conformation by mAbs
Type of data	X-ray crystal structures, surface plasmon resonance studies (SPR), and generation of mAbs with defined reactivity
How data was acquired	X-ray diffraction data were collected at Shanghai Synchrotron Radiation Facility
SPR data was recorded on a CM5 chip with a Biacore3000™ (GE Healthcare Life Sciences)
Data format	Processed
Experimental factors	Recombinant proteins and monoclonal antibodies were affinity purified to high homogeneity before use.
Experimental features	Kinetic rate constants for the interaction between immobilized anti-uPAR mAbs and recombinant uPAR mutants were determined by SPR, the structure of the mAb·uPAR complex was determined by X-ray crystallography
Data source location	Not applicable
Data accessibility	The data is available from the related publication by Zhao et al. (http://www.ncbi.nlm.nih.gov/pubmed/25659907), from the patent (WO 2013/020898 A12013) and the structures deposited in the Protein Data Bank (entries 4QTH and 4QTI).

**Value of the data**

• Defines the structure of a closed, active conformation of native uPAR^wt^ without covalent modifications;
• defines a topographic epitope landscape on uPAR for 6 different bins of anti-uPAR mAbs;
• establish that occupancy of the Vn-binding site by mAbs drives uPAR into to its closed conformation;
• data defining this interdomain flexibility are important for functional studies on uPAR biology;
• and for the future design of uPAR-targeted intervention studies in human disease [1,7-9].

## 1 Data and experimental design

**Fig. 1:**
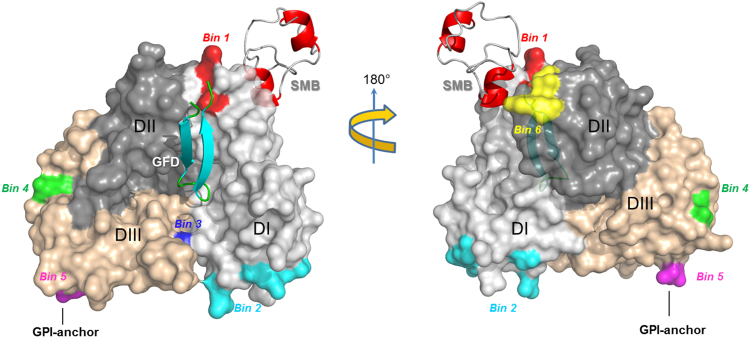
Topographic landscape on human uPAR for different mAb epitope bins. The crystal structure of human uPAR is shown in a surface representation (3BT1) with the individual LU domains color coded; DI (light gray), DII (dark gray) and DIII (wheat). The receptor-binding domains of the natural ligands are shown as cartoon representation i.e. the serine protease urokinase (GFD) and the matrix protein vitronectin (SMB). The defined epitope bins are highlighted by colors: **BIN 1** in red representing mAbs R3, R21 and VIM-5; **BIN 2** in cyan representing mAbs R5, R9, mR1 and R20; **BIN 3** in blue representing mAb H2; **BIN 4** in green representing mAbs R4 and R8; **BIN 5** in magenta representing mAbs R2, R24 and ATN-658; and **BIN 6** in yellow representing mAbs 8B12 and 19.10. The identities of the hot-spot residues in the individual bins are provided in Table 1 in the original publication [5].

We have developed an experimental platform to map the functional and structural epitopes on uPAR for anti-uPAR monoclonal antibodies by combining surface plasmon resonance studies (SPR) with X-ray crystallography and the data is summarized in Fig. 1. We expressed the recombinant uPAR variants in *Drosophila* S2-cells as soluble and secreted proteins by deleting the C-terminal signal peptide required for membrane tethering by a glycosyl-phosphatidylinositol anchor [10]. The choice of this particular host organism for recombinant uPAR expression is dual. First, the transfection efficacy of the S2 cells is extremely high rendering laborious sub-cloning superfluous in most cases. Second, the simple and homogenous N-linked glycosylation patters provided by these cells are advantageous for crystallization [11].

The initial mapping of the epitopes for new anti-PAR mAbs was performed by immobilizing the antibody in question on a CM5 sensor chip™ (GE Helthcare) with conventional amide chemistry (EDC/NHS). First, the kinetics rate constants (*k*_*on*_ and *k*_*off*_) for the mAb**·**uPAR interactions were determined at low surface densities to minimize possible confounding effects from mass transport limitations. Second, the domain reactivity of these mAbs was established by measuring the binding to intact uPAR, uPAR DI, and uPAR DIIDIII. Third, epitope binning was performed by measuring whether a second anti-uPAR mAb can bind to the uPAR captured by the immobilized mAb. Fourth, a complete single-site epitope mapping was performed by measuring the kinetics between the immobilized mAb and a library of purified single site uPAR mutants produced in S2 cells [3,6].

Those anti-uPAR mAbs with unique and relevant functional and/or structural properties were then selected for further X-ray crystallography studies to provide a high-resolution structure of the topography of the mAb uPAR binding interface and define which of the uPAR conformations is selected by that particular mAb. The power of this approach is illustrated by the apparent convergent evolution of the three-dimensional hot-spot configuration of the binding interface between the native biologic ligand (vitronectin) and the surrogate ligand (mAb 8B12) – see Fig. 5 in the original publication [5]. This relationship is not evident from direct sequence alignments of the primary structures of the mAbs in question and the natural ligand vitronectin. The summary of the topographic epitope landscape on human uPAR is illustrated below in Fig. 1 and this information provides an important working tool for selecting those particular mAbs that are optimal for a given biological experiment and the knowledge of the corresponding hot-spot residues on uPAR may in some case also provide the ideal negative control uPAR mutant for such intervention studies. The general utility of this “toolbox”, which is providing data on epitope-mapped anti-uPAR mAbs, is evident from the following example. How do you choose the optimal reagent for functional blocking of uPAR-mediated effects on cell adhesion? If mAbs from epitope bin 1 (*i.e.* R3 or R21) are selected as intervention agents they will indeed abrogate uPAR-mediated adhesion on vitronectin in conditions with very low levels of uPA [3,12,13], but this effect will critically depend on the level of uPAR-occupancy with uPA as these mAbs will not bind uPA**·**uPAR complexes [5]. Another confounding factor in such studies is the observation that uPA-binding as such increases cell migration [4,14]. These complicating factors are, nonetheless, minimized if anti-uPAR mAbs from bin 6 are selected as intervention agents, as mAbs from this particular epitope bin (*i.e.* 8B12 or 19.10) inhibit vitronectin binding and uPAR-mediated cell adhesion even under conditions when the receptor is completely saturated with uPA [5].

## 2 Materials and methods

### 2.1 Recombinant protein production and design of a permanently closed uPAR variant

*Drosophila* S2 cells have proven to be an excellent host organism for heterologous expression of recombinant human and mouse uPAR both with a view to biophysical structure determination by X-ray crystallography [5,15–21], hydrogen–deuterium exchange [2,20,22] or small angle X-ray scattering [2] and with a view to functional studies by surface plasmon resonance [6,18,23] and microtiter-based time-resolved fluorescence [24]. The recombinant uPAR protein is secreted from the transfected S2 cells to the harvest fluid due to the omission in the expression vector of a C-terminal signal sequence entailing the post-translational addition of a glycosyl-phosphatidylinositol membrane anchor [10]. For a detailed experimental protocol on uPAR expression in S2 cells please consult [11]. The secreted uPAR can be affinity purified with CNBr-activated Sepharose™ coupled to specific anti-uPAR mAbs (mAb R2 for human uPAR and mAb KOR-1 for mouse uPAR) [6,25], the uPAR-binding endogenous ligand uPA or its receptor-binding fragment ATF [26,27], or a small high-affinity peptide antagonist developed by combinatorial chemistry [25,28]. These protocols generally yields>2 mg purified uPAR per liter harvest fluid. Relatively gentle elution conditions can be applied in the case of affinity purification with uPA-coupled Sepharose due to the relatively high pKa of 5.8 for the uPA*·*uPAR interaction [25,29].

If the protein in question is difficult to express or the S2 cells do not thrive, very low expression yields are obtained and this is often associated with co-purification of a contaminating endogenous *Drosophila-*protein (imaginal disk growth factor protein 2, GI:45476987). The downside of adding 0.5 mM Cu^2+^, required for activation of the metallothionein promoter, is that it may promote precipitation of proteins and/or lipids. From a practical point of view it is therefore important to add 20 mM EDTA to the harvest fluid and thoroughly removed all microparticles by centrifugation or filtration to improve the life-time of the affinity column by avoiding clotting. A second sensible precaution is to reverse flow between sample application and the washing and elution.

The successful application of S2 cell expression for recombinant uPAR production has furthermore evolved into a new protocol for expression of other LU-domain containing proteins using the third LU-domain in uPAR (DIII) as fusion partner and the anti-uPAR mAb R2 for detection, optimization of expression yields, and purification tag [30]. The DIII fusion tag can be placed N-terminally or C-terminally dependent of the target protein in question [31,32]. If uPAR DIII is used as an N-terminal tag it is advantageous to mutate Arg^281^ in the linker to a Gly to prevent unintentional cleavage at this site during enterokinase-mediated excision of the target protein (unpublished).

Two different avenues have been explored to design a constitutively active uPAR that preferentially populates the closed state, which is induced as a consequence of the high-affinity binding of uPA [2,3]. The first approach was to introduce a disulfide bond between His^47^ and Asn^259^, which covalently tethers uPAR DI and DIII [19,33]. The second approach was to make a GFD-uPAR-Fc chimera in which the N-terminal receptor-binding growth factor-like domain of uPA (denoted GFD) is fused in-line via a linker to the N-terminus of uPAR, which again is followed by a second linker and the Fc-region of IgG [patent; WO 2013/020898 A12013]. This design entails a uni-molecular saturation mechanism permanently driving uPAR into its closed conformation. The corresponding protein chimera was expressed by Chinese Hamster ovary (CHO) cells and was purified from the supernatant by conventional protein A affinity chromatography and was used for immunization giving rise to the mAbs denoted 13F6, 8B12, 10H6, 13D11, 10.10, AL6, AL38, and BE18 [patent; WO 2013/020898 A12013].

### 2.2 Real-time binding interaction by surface plasmon resonance

To map the functional epitope on uPAR for various anti-uPAR mAbs to “single site resolution” the kinetic rate constants (*k*_*on*_ and *k*_*off*_) for the mAb•uPAR interaction in real-time by surface plasmon resonance with a Biacore 3000™ was determined. To eliminate avidity effects and mass transport limitations, the mAbs were covalently immobilized on a CM5™chip at a density of 5–6 pmol/mm^2^ by amine chemistry and >250 uPAR mutants were subsequently tested as analytes in a multi-cycle format. Each uPAR mutant was tested in a block comprising one buffer run, three runs of a 3-fold dilution of uPAR spanning 90 nM, 30, nM and 10 nM, and one repeat analysis of 30 nM uPAR in the end of the block to demonstrate reproducibility. The mAbs were regenerated between each cycle by two separate injections of 0.1 M acetic acid in 0.5 M NaCl and 20 mM H_3_PO_4_. Generally these regeneration conditions were well-tolerated by the immobilized mAbs allowing the run of 25–100 blocks to be completed before a new immobilization was required. Quality checks consisted of running uPAR^wt^ every 15–20 block to detect any impairment in performance. The running buffer was 10 mM Hepes (pH 7.4), 150 mM NaCl, 3 mM EDTA and 0.005% (*v*/*v*) P-20 and binding interactions were conducted at 20 °C at a flowrate of 50 µl/min.

### 2.3 X-ray crystallography

Fab fragment of mAb 8B12 was generated by digestion with papain as described in the original publication. The liberated Fab fragments was purified by Protein A column and anion exchange chromatography using a MonoQ column. The Fab fragment was then mixed with recombinant human uPAR in a molar ratio of 1:1.5. The complex was incubated at room temperature for half an hour, and purified by size exclusion chromatography (Superdex 75 h 10/30, Pharmacia Biotech).

For crystallization, the Fab or the protein complex was dialyzed into a buffer of 10 mM Tris–HCl pH 7.4 without salt. All crystals were obtained by mixing the protein solution with an equal amount of reservoir solution by the sitting-drop vapor-diffusion method at room temperature. Micro-seeding was applied to improve the quality of the complex crystals. For data collection, the crystals of Fab fragment and the complex were flash-frozen in liquid nitrogen in the reservoir solution containing 10% DMSO and 20% Glycerol, respectively.

X-ray diffraction data was collected at the Shanghai Synchrotron Radiation Facility and were processed using the HKL2000 suite. All crystal structures were solved by molecular replacement using Molrep from the CCP4 program suite. The crystal structure of 8B12 Fab was solved using murine 125-2H Fab (2VXT) as the initial search model. The refine 8B12 Fab fragment structure was used as the initial search model for the structure determination of the protein complex. The position of uPAR in the complex was found using the PDB entry 2FD6 by fixing the position of 8B12 Fab. Both the 8B12 Fab and the complex were manual adjusted by COOT and refined by REFMAC, were deposited in PDB with the code 4QTH and 4QTI, respectively.
